# Transport of Apolipoprotein B-Containing Lipoproteins through Endothelial Cells Is Associated with Apolipoprotein E-Carrying HDL-Like Particle Formation

**DOI:** 10.3390/ijms19113593

**Published:** 2018-11-14

**Authors:** Hong Yang, Ningya Zhang, Emmanuel U. Okoro, Zhongmao Guo

**Affiliations:** Department of Microbiology, Immunology and Physiology, Meharry Medical College, Nashville, TN 37208, USA; nzhang@mmc.edu (N.Z.); eokoro@mmc.edu (E.U.O.)

**Keywords:** transendothelial transport, apolipoprotein B-containing lipoprotein, high-density lipoprotein formation, apolipoprotein E, mouse aortic endothelial cells

## Abstract

Passage of apolipoprotein B-containing lipoproteins (apoB-LPs), i.e., triglyceride-rich lipoproteins (TRLs), intermediate-density lipoproteins (IDLs), and low-density lipoproteins (LDLs), through the endothelial monolayer occurs in normal and atherosclerotic arteries. Among these lipoproteins, TRLs and IDLs are apoE-rich apoB-LPs (E/B-LPs). Recycling of TRL-associated apoE has been shown to form apoE-carrying high-density lipoprotein (HDL)-like (HDL_E_) particles in many types of cells. The current report studied the formation of HDL_E_ particles by transcytosis of apoB-LPs through mouse aortic endothelial cells (MAECs). Our data indicated that passage of radiolabeled apoB-LPs, rich or poor in apoE, through the MAEC monolayer is inhibited by filipin and unlabeled competitor lipoproteins, suggesting that MAECs transport apoB-LPs via a caveolae-mediated pathway. The cholesterol and apoE in the cell-untreated E/B-LPs, TRLs, IDLs, and LDLs distributed primarily in the low-density (LD) fractions (*d* ≤ 1.063). A substantial portion of the cholesterol and apoE that passed through the MAEC monolayer was allotted into the high-density (HD) (*d* > 1.063) fractions. In contrast, apoB was detectable only in the LD fractions before or after apoB-LPs were incubated with the MAEC monolayer, suggesting that apoB-LPs pass through the MAEC monolayer in the forms of apoB-containing LD particles and apoE-containing HD particles.

## 1. Introduction

Apolipoprotein B-containing lipoproteins (apoB-LPs) are generated in the liver and intestine, and converted to various sizes of remnants in circulation. Based on their buoyant density, apoB-LPs are classically divided into four major subclasses, designated as chylomicrons, very low-density lipoproteins (VLDLs), intermediate-density lipoproteins (IDLs), and low-density lipoproteins (LDLs). Among these lipoproteins, LDLs are poor in apoE and triglycerides (TGs), while the other three subclasses are apoE-rich lipoproteins. The TG-rich lipoproteins (TRLs) are functionally defined as the fraction of plasma lipoproteins at a density of <1.006 g/mL [1], which includes chylomicrons and VLDLs. The intestine-originated TRLs chylomicrons contain apoB48 and 85–92% TGs, while VLDLs, the liver-originated TRLs, contain apoB100 and 50–55% TGs [2]. When circulating in the blood, chylomicrons, and VLDLs interact with lipoprotein lipase, which converts chylomicron to VLDL- and IDL-sized chylomicron remnants, and converts VLDLs to IDLs and LDLs by removing TGs from the TRLs [2]. Due to the short half-life of chylomicrons and their remnants, TRLs in the blood under fasting conditions are mainly apoB100-carrying particles.

Recycling of TRL-associated apoE reportedly resulted in formation of apoE-rich high-density lipoproteins (HDLs). Specifically, the internalized TRLs disintegrate in early endosomes. A portion of apoE escapes lysosomal degradation and is recycled back to the plasma membrane, followed by resecretion [3]. Recycling of TRL-associated apoE in hepatocytes provides the plasma with apoE-rich HDLs [4,5], which in turn donates apoE to plasma TRLs. Impaired apoE recycling in hepatocytes, therefore, induces low plasma HDL and high TRL levels [3]. Recycling of apoE in macrophages is associated also with HDL-like particle formation and cholesterol efflux [6]. Impaired apoE recycling in macrophages may lead to foam cell formation. In addition, deficiency in apoE recycling in neuronal cells has been implicated in the onset of Alzheimer’s disease [7].

Passage of plasma apoB-LPs to the subendothelial space occurs in the normal arteries and is accelerated substantially in atherosclerotic lesions [8,9]. Compelling evidence suggests that lipoproteins may pass over the monolayer endothelium via paracellular and transcellular pathways [10,11,12]. Transcellular transport, i.e., transcytosis, includes three steps: Endocytosis; intracellular trafficking; and exocytosis. Knockout of mouse caveolin-1 [13], the major protein component of caveolae, and knockout of scavenger receptor BI (SR-BI) [14] or activin-like kinase 1 (ALK1) [15] have been shown to inhibit the endocytosis of LDLs in endothelial cells. Both SR-BI and ALK1 are caveolae-located receptors. In addition, knockout of mouse caveolin-1 diminished LDL transport to the arterial wall [16] and inhibited atherosclerosis [17], suggesting that endothelial uptake of LDLs is an ALK1- and SR-BI-mediated endocytosis that uses caveolar vesicles to bring LDL particles into cells. The caveolar endocytic vesicles deliver contents to early endosomes [18], where internalized cargos are sorted either to lysosomes for degradation or recycling endosomes for resecretion. Studies using in vitro transwell models showed that native LDL particles endocytosed from the apical (AP) side of endothelial cells are not delivered to lysosomes, but are transported to the basolateral (BL) side as a holoparticle [14]. This is consistent with the finding that VLDL- and LDL-sized particles isolated from human [19,20] and rabbit [8] arteries contain intact apoB and apoE.

For the first time, the present report demonstrated that apoE-rich apoB-LPs (E/B-LPs), i.e., TRLS and IDLs, passed the mouse aortic endothelial cell (MAEC) monolayer via a transcellular process as LDLs do and that transcytosis of E/B-LPs and LDLs through the MAEC monolayer was associated with formation of apoE-carrying HDL-like (HDL_E_) particles. Transendothelial transport of E/B-LPs forms more HDL_E_-like particles than transport of LDLs.

## 2. Results

### 2.1. Transport of apoB-LPs through the MAEC Monolayer Is Competition-Dependent and Filipin Inhibitable

**Figure 1:**
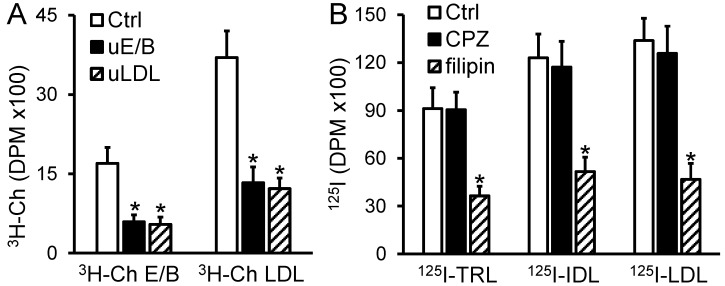
Transendothelial transport of radiolabeled apoB-LPs is inhibited by filipin and unlabeled competitor lipoproteins. (**A**): The AP side of the MAEC monolayer was incubated with 20 µg/mL (35,000 DPM) of ^3^H-Ch-labeled E/B-LPs (^3^H-Ch E/B) or ^3^H-Ch-labeled LDLs (^3^H-Ch LDL) in the presence of 800 µg/mL of unlabeled E/B-LPs (uE/B) or unlabeled LDLs (uLDLs) or in the absence of unlabeled lipoproteins as a control (Ctrl) for 24 h. (**B**): The AP side of the MAEC monolayer was pre-incubated with 20 µg/mL chlorpromazine, 5 µg/mL filipin, or 1 µL/mL dimethyl sulfoxide as a Ctrl for 1 h, followed by incubation with 20 µg/mL (167,000 DPM) of ^125^I-labeled TRLs, IDLs, or LDLs for 24 h. The radioactivity in the BL medium were counted. Values represent the mean ± SE of 3–5 experiments; * *p* < 0.05 vs. Ctrl.

Though transcytosis of LDLs through endothelial cells has been demonstrated in vitro and in vivo [12,13,14], very little is known about how E/B-LPs, i.e., TRLs and IDLs, pass through the monolayer endothelium. The current study examined the transport of LDLs and E/B-LPs through the MAEC monolayer in a transwell system. After incubation of 20 µg/mL ^3^H-cholesterol (^3^H-Ch)-labeled E/B-LPs or LDLs with the AP side of the MAEC monolayer for 24 h, an average of 5.4% of E/B-LP-associated ^3^H-Ch and 11.3% of LDLS-associated ^3^H-Ch were transported to the BL chamber (Figure 1A). Addition of 800 µg/mL unlabeled E/B-LPs (uE/B-LPs) into the AP chamber reduced E/B-LP- and LDL-associated ^3^H-Ch transport by 75% and 68%, respectively (Figure 1A). The same amount of unlabeled LDLs (uLDLs) reduced E/B-LP- and LDL-associated ^3^H-Ch transport by 71% and 63%, respectively (Figure 1A). The competition of uE/B-LPs and uLDLs with their radiolabeled counterparts suggests that apoB-LPs, whether rich or poor in apoE, pass through the MAEC monolayer via a specific pathway-mediated transcellular transport rather than a simple diffusion across the paracellular gaps. The mutual competitive inhibition between E/B-LPs and LDLs suggests that these lipoproteins share a common transport pathway.

To confirm the transcellular transport of apoB-LPs in our transwell model, we studied the effect of chlorpromazine and filipin on the transport of ^125^I-labeld TRLs, IDLs, and LDLs through the MAEC monolayer. Chlorpromazine depletes adaptor proteins and clathrin from the plasma membrane and inhibits clathrin-mediated endocytosis, while filipin binds to cell surface cholesterol and inhibits caveolae-dependent endocytosis [21]. In this study, the AP side of the MAEC monolayer was pretreated with 20 µg/mL chlorpromazine, 5 µg/mL filipin, or a medium vehicle for 1 h, followed by treatment with 167,000 DPM (~20 µg/mL) of ^125^I-labeled TRLs, IDLs, or LDLs. After 24 h of incubation, an average of 9115 DPM of ^125^I-labeled TRLs, 12,296 DPM of ^125^I-labeled IDLs, and 13,379 DPM of ^125^I-labeled LDLs were transported to the BL chamber through the MAEC monolayer under control conditions (Figure 1B). Filipin reduced TRL, IDL, and LDL transport by 60%, 58%, and 65%, respectively (Figure 1B). In contrast, chlorpromazine did not significantly inhibit the transendothelial transport of these apoB-LPs. This observation is consistent with previous reports showing that transcytosis of LDLs through arterial endothelial cells is mediated primarily via a caveolae-mediated pathway [13,16].

### 2.2. Transendothelial Transport of apoB-LPs Did Not Alter Their Free/Total Cholesterol Ratio

**Figure 2:**
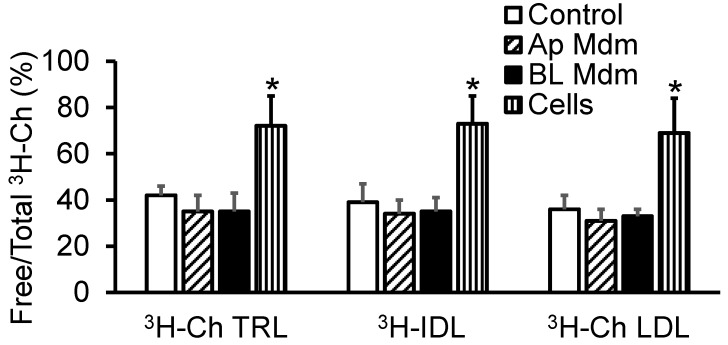
The free/total cholesterol ratio of transported apoB-LPs remains unchanged. The AP side of the MAEC monolayer was incubated with 20 µg/mL (35,000 DPM) of ^3^H-Ch-labeld TRLs, IDLs, or LDLs for 24 h. The free ^3^H-Ch and esterified ^3^H-Ch in the AP and BL media and cell lysates, as well as in the cell-untreated TRLs, IDLs, and LDLs (control), were separated by digitonin precipitation and assessed by scintillation counting. The percentage of free to total ^3^H-Ch was calculated as follows: (free ^3^H-Ch/(esterified ^3^H-Ch + free ^3^H-Ch)) × 100. Values represent the mean ± SE of three experiments; * *p* < 0.05 vs. control.

In many cell types, such as macrophages and hepatocytes, the cholesterol ester moiety of the internalized apoB-LPs is sorted to lysosomes and hydrolyzed by lysosomal acid lipase. To assess whether cholesterol ester hydrolysis occurs along with the transcytosis of apoB-LPs through endothelial cells, we determined the free/total ^3^H-Ch ratio in these lipoproteins before and after incubating them with the MAEC monolayer. Data in Figure 2 show that ~40% of the ^3^H-Ch in the cell-untreated TRLs, IDLs, and LDLs exists in unesterified form. Incubation of ^3^H-Ch-labeled TRLs, IDLs, and LDLs with the MAEC monolayer did not elevate, but slightly reduced, the percentage of free/total ^3^H-Ch in the AP and BL media. This suggests that transcytosis of apoB-LPs through MAECs did not result in cholesterol ester hydrolysis. The data in Figure 2 also suggest that the ^3^H-Ch accumulated in MAECs exists primarily in unesterified form. Specifically, ~71%, 72%, and 69% of the ^3^H-Ch in the MAECs treated with ^3^H-Ch TRLs, IDLs, and LDLs, respectively, were free cholesterol.

### 2.3. Transendothelial Transport of apoB-LPs Resulted in Cholesterol Redistribution among Lipoprotein Fractions

**Figure 3:**
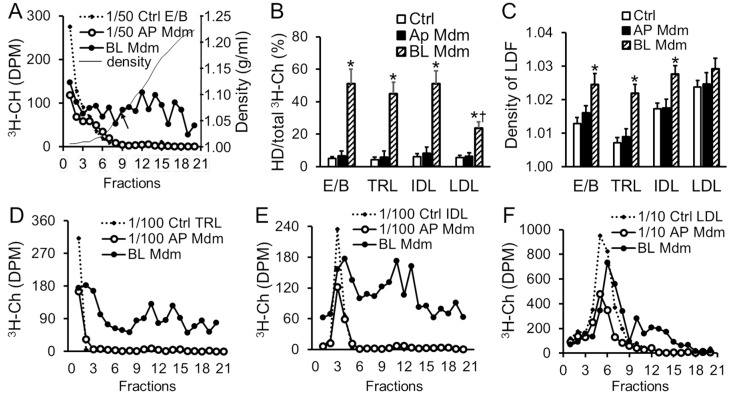
Transendothelial transport of apoB-LPs resulted in cholesterol redistribution among lipoprotein fractions. The AP side of the MAEC monolayer was incubated with 20 µg/mL of ^3^H-Ch-labeld E/B-LPs (E/B) (**A**), TRLs (**D**), IDLs (**E**), or LDLs (**F**); the BL side was incubated with medium alone for 24 h. The AP and BL media (Mdm), and 20 µg of cell-untreated control ^3^H-Ch-labeled lipoproteins (i.e., Ctrl E/B, TRL, IDL, or LDL), were subjected to DG ultracentrifugation. Twenty fractions were collected for determination of density and radioactivity. The level of ^3^H-Ch was expressed by radioactivity counts: 1/100, 1/50, 1/10 (radioactivity counts in Ctrl lipoproteins and AP medium were divided by 100, 50, or 10). A representative density curve was presented in panel **A**. The arrow pointing to the density curve indicated *d* = 1.063 g/mL. (**B**): The level of ^3^H-Ch in the HD fractions of the AP and BL media and control lipoproteins (Ctrl) was expressed as the % of the HD ^3^H-Ch in the total ^3^H-Ch (HD/total ^3^H-Ch), which was calculated as follows: (The radioactivity in fractions 10–20/the radioactivity in fractions 1–20) × 100. (**C**): The average density of the lipoproteins in low density fractions (LDF), i.e., fractions 1–9, was calculated as follows: ($\sum_{i=1}^{9}DiRi/\sum_{i=1}^{9}Ri$), where D and R are the hydrated density and radioactivity count of each fraction, respectively. Values represent the mean ± SE of four experiments; * *p* < 0.05 vs. the Ctrl EB-LPs, TRLs, IDLs, or LDLs; ^†^ *p* < 0.05 vs. BL medium containing ^3^H-Ch EB-LPs, TRLs, or IDLs.

To study whether transcytosis of apoB-LPs through the MAEC monolayer generates HDL-like particles, we analyzed the density distribution of lipoprotein-associated ^3^H-Ch using a discontinuous density-gradient (DG) ultracentrifugation. As the density curve in Figure 3A indicated, the 1st, 4th, 9th, and 19th fractions of the centrifuged gradient solution have densities of 1.006 g/mL, 1.019 g/mL, 1.063 g/mL, and 1.21 g/mL, respectively. This suggests that the DG ultracentrifugation protocol used in this study is able to distribute TRLs to the 1st fraction, IDLs to the 2nd–4th fractions, LDLs to 5th–9th fractions, and HDLs to 10th–20th fractions of the solution. We designated fractions 1–9 as low-density (LD) fractions and fractions 10–20 as high-density (HD) fractions.

The radioactivity distribution curve in Figure 3A illustrates that the ^3^H-Ch of cell-untreated Ctrl E/B-LPs distributes primarily in fractions 1–4. Transport of E/B-LPs through the MAEC monolayer induced ^3^H-Ch redistribution among density fractions. Specifically, the radioactivity in the BL medium exhibited multiple peaks in both the LD and HD fractions. About 51% of the radioactivity presented in the HD fractions. The HD/total ^3^H-Ch ratio was significantly greater in the BL medium than in the cell-untreated E/B-LPs (Figure 3A,B). This suggests that transcytosis of E/B-LPs through the MAECs is accompanied by formation of HDL-like particles. In the LD fractions of the BL medium, most of the radioactivity distributed in IDLs and LDLs (i.e., fractions 2–9), which led to a significant increase in the average density of the LD fractions (Figure 3C). Incubation of E/B-LPs with the MAEC monolayer in the AP chamber slightly increased the average density of LD fractions and the HD/total ^3^H-Ch ratio in the AP medium; however, the difference between the AP medium and cell-untreated E/B-LPs was not statistically significant (Figure 3B,C).

Next, we studied the TRL and IDL components of E/B-LPs separately for cholesterol redistribution induced by transendothelial transport. As Figure 3D,E showed, ~90% of the radioactivity in the cell-untreated TRLs presented in the 1st fraction, and ~90% of the radioactivity in the cell-untreated IDLs presented in fractions 2–4, with a peak in fraction 3. Similar to E/B-LPs, both TRLs and IDLs generated HDL-like particles when they passed through the MAEC monolayer. Nearly half of the TRL and IDL ^3^H-Ch that had passed through the MAEC monolayer distributed to HD fractions. The HD/total ^3^H-Ch ratios in the BL medium were significantly greater than ratios in the cell-untreated TRLs and IDLs (Figure 3B). In the LD fraction of the BL medium, a portion of TRL ^3^H-Ch shifted to IDL and LDL fractions (Figure 3D), and a portion of IDL ^3^H-Ch shifted to the LDL fractions (Figure 3E). These led to a significant increase in the average density of the LD fractions of the BL medium, as compared with the cell-untreated TRLs and IDLs (Figure 3C). Incubation of TRLs or IDLs with the MAEC monolayer in the AP chamber did not significantly alter the distribution of ^3^H-Ch among the density fractions in AP medium (Figure 3D,E). The average density of LD fractions (Figure 3C) and the HD/total ^3^H-Ch ratio (Figure 3B) were not significantly higher in the AP medium than in cell-untreated TRLs and IDLs.

Finally, we studied the effect of transendothelial transport on the distribution of LDL-associated ^3^H-Ch. The radioactivity in the LDLs that were not incubated with the MAEC monolayer exhibited a single peak in fraction 5 (*d* = 1.039) (Figure 3F). Incubation of ^3^H-Ch-labeled LDLs with the MAEC monolayer did not significantly alter the density distribution of ^3^H-Ch in the AP medium, namely a single radioactivity peak distributed at *d* = 1.039 g/mL (Figure 3F). In contrast, the radioactivity in the BL medium showed multiple peaks, and ~26% of the radioactivity presented in the HD fractions (Figure 3F,B). This suggests that transcytosis of LDLs through MAECs also forms HDL-like particles, though it is less productive than transcytosis of E/B-LPs, TRLs, or IDLs (Figure 3B). In addition, the position of the radioactivity peak in the LD fractions of the BL medium shifted from *d* = 1.039 g/mL to *d* = 1.041 g/mL (Figure 3F).

### 2.4. Transendothelial Transport of apoB-LPs Resulted in apoE Redistribution among Lipoprotein Fractions

**Figure 4:**
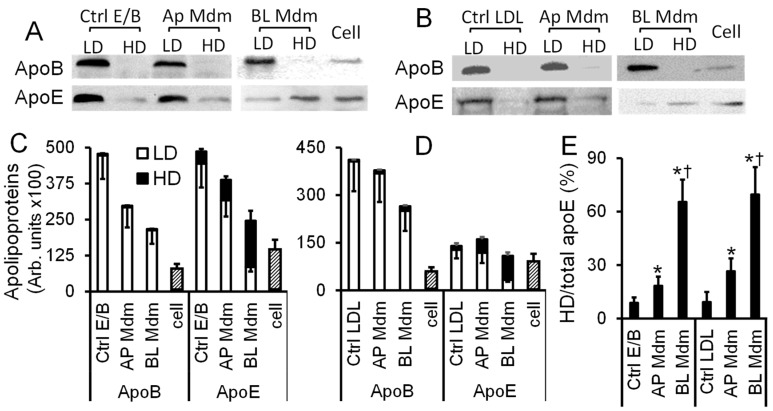
Transendothelial transport of apoB-LPs induced redistribution of apoE, but not apoB, among lipoprotein fractions. The AP side of the MAEC monolayer was incubated with 20 µg/mL of E/B-LPs (E/B) (**A**,**C**) or LDLs (**B**,**D**); the BL side was incubated with medium alone for 24 h. The AP and BL media, and 20 µg of cell-untreated Ctrl E/B or LDLs, were subjected to DG ultracentrifugation. Fractions 1–9 and 10–20 were pooled separately and designated as LD and HD fractions, respectively. Proteins in the LD and HD fractions and the cell lysate were precipitated with 75% methanol for Western blot analysis of apoB and apoE. The protein in the BL medium was pooled from six BL chambers. The level of apoE was expressed by the immunoblot band intensity (**C**,**D**). (**E**): The % of apoE in the HD fraction to the total apoE (HD/total apoE) in the AP and BL media, and the Ctrl E/B and LDLs, was calculated as follows: (apoE in the HD fraction/(apoE in the HD fraction + apoE in the LD fraction)) × 100. Values represent the mean ± SE of three independent experiments; * *p* < 0.05 vs. the Ctrl EB-LPs or LDLs, and ^†^ *p* < 0.05 vs. AP medium.

It has been reported that the apoE internalized with TRLs, along with a portion of lipids, dissociates from the apoB-containing lipid core and exits hepatocytes and macrophages as HDL_E_ particles [6]. To explore whether dissociation of apoE from the apoB-containing lipid core also occurs during transendothelial transport of E/B-LPs and LDLs, we analyzed the density distribution of apoE in these lipoproteins before and after incubation with the MAEC monolayer. Our initial experiments using Western blot analysis showed that apoB protein was detectable only in the LD fraction, but not in the HD fraction of the cell-untreated E/B-LPs and LDLs (Figure 4A,B). Incubation of E/B-LPs and LDLs with the MAEC monolayer in the AP chamber did not induce apoB redistribution among density fractions; apoB was detectable only in the LD fraction of the AP and BL media (Figure 4A–D).

In contrast, incubation of E/B-LPs and LDLs with the MAEC monolayer resulted in an apoE shift from the LD to HD fractions. Specifically, most of the apoE in the cell-untreated E/B-LPs and LDLs was present in the LD fraction, and only a small portion was fond in the HD fraction (Figure 4A–D). Incubation of these lipoproteins with the MAEC monolayer resulted in apoE redistribution among density fractions. Although the level of apoE is lower in LDLs than in E/B-LPs, the percentage of apoE in the HD fraction to the total apoE (HD/total apoE) in these lipoproteins was comparable (Figure 4E). Specifically, the HD/total apoE in both cell-untreated E/B-LPs and LDLs was <10%. After incubation of E/B-LPs with the MAEC monolayer in the AP chamber, ~18% and 65% of the apoE was found in the HD fractions in the AP and BL media, respectively (Figure 4E). Similarly, after incubation of LDLs with the MAEC monolayer, ~26% and 70% of the apoE was found in the HD fractions in the AP and BL media, respectively (Figure 4E). In addition, incubation of the MAEC monolayer with E/B-LPs and LDLs resulted in intracellular accumulation of apoB and apoE, i.e., a substantial apoB and apoE accumulation in the E/B-LP- and LDL-treated cells (Figure 4A–D).

**Figure 5:**
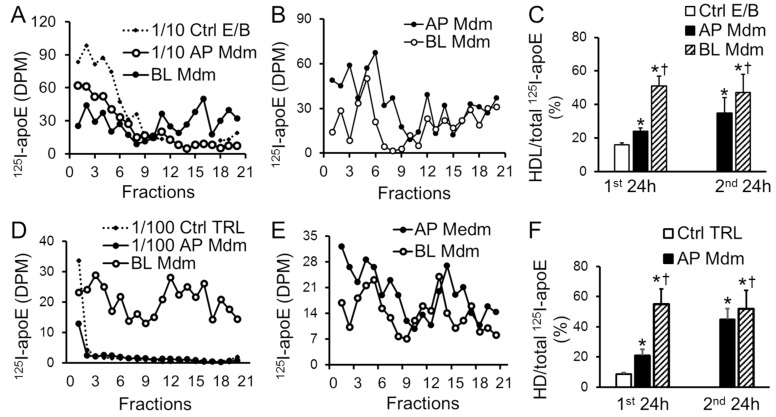
Transendothelial transport of apoB-LPs resulted in apoE redistribution among lipoprotein fractions. The AP side of the MAEC monolayer was incubated with ^125^I-apoE E/B-LPs (E/B) (**A**–**C**) or TRLs (**D**–**F**); the BL side was treated with medium alone for 24 h (1st 24 h). (**B**,**E**): The MAEC monolayer, treated as above, was washed with heparin solution (**B**), or low ionic strength solution (**E**), and followed by incubation with fresh medium for another 24 h (2nd 24 h). The AP and BL media collected from the 1st 24 h and the 2nd 24 h incubations, as well as the cell-untreated Ctrl ^125^I-apoE E/B-LPs and TRLs, were subjected to DG ultracentrifugation. Twenty fractions were collected for determination of radioactivity. (**C**,**F**): The % of the HD fraction ^125^I-apoE in the total ^125^I-apoE of the AP and BL media, and the Ctrl E/B-LPs and TRLs (HD/total ^125^I-apoE) was calculated using the following equation: (The radioactivity in fractions 10–20/the radioactivity in fractions 1–20) × 100. Values represent the mean ± SE of three experiments; * *p* < 0.05 vs. the cell-untreated Ctrl E/B-LPs or TRLs, and ^†^ *p* < 0.05 vs. AP medium.

In the following set of experiments, ^125^I-apoE was incorporated into E/B-LPs and TRLs. The radioactivity of the ^125^I-apoE-containing E/B-LPs (^125^I-apoE E/B-LPs) that were not incubated with the MAEC monolayer was distributed primarily in fractions 1–4 (Figure 5A), while most of the radioactivity in the cell-untreated ^125^I-apoE-containing TRLs (^125^I-apoE TRLs) was distributed primarily in the 1st fraction (Figure 5D). Transport of these lipoproteins through the MAEC monolayer resulted in a portion of ^125^I-apoE shift from the LD fractions to the HD fractions. Specifically, ~50% of the E/B-LP- and TRL-associated ^125^I-apoE that had passed through the MAEC monolayer presented in the HD fractions of the BL medium (Figure 5C,F). This suggests that nearly one-half of the ^125^I-apoE in the BL medium exists as HDL-sized lipidated particles. The data in Figure 5C,F also indicate that the HD/total ^125^I-apoE ratio in the AP medium is significantly greater than that in the cell-untreated E/B-LPs and TRLs.

Next, we studied the resecretion of ^125^I-apoE from the MAECs pretreated with ^125^I-apoE E/B-LPs or TRLs. Data in Figure 5B,E indicated that ^125^I-apoE was resecreted to both the AP and BL sides, and the resecreted ^125^I-apoE was distributed in both the LD and HD fractions. Specifically, ~35% and 54% of the ^125^I-apoE resecreted from the E/B-LP-treated cells were presented in the HD fractions of the AP and BL media, respectively; ~45% and 52% of the ^125^I-apoE resecreted from the TRL-treated cells were presented in the HD fractions of the AP and BL media, respectively (Figure 5F).

## 3. Discussion

For the first time, the current report provided evidence that transcytosis of E/B-LPs and LDLs through MAECs resulted in disintegration of these lipoprotein particles. Specifically, we observed that a substantial portion of apoE and cholesterol of the E/B-LPs and LDLs that passed through the MAEC monolayer presented in the HD fractions, while apoB, along with rest of the transported apoE and cholesterol, presented in the LD fractions. It is known that LDLs compared with E/B-LPs, i.e., TRLs and IDLs, contain less apoE, especially the smallest LDL particles, which entirely lost apoE [6]. Our data indicate that the percentage of cholesterol distributed to the HD fractions is positively related to the apoE protein level in these fractions. Only a small portion of LDL cholesterol that passed the MAEC monolayer was distributed to the HD fractions. In contrast, more than half of the transported E/B-LP cholesterol was allotted to the HD fractions. Dissociation of apoE from endocytosed E/B-LP is not cell-specific, as it has been reported in other cell types, such as fibroblasts [22], macrophages [4,6], hepatocytes [4], and Chinese Hamster ovary cells [23]. The apoE resecreted from those cells was floated with lipids or presented in a relatively lipid-poor state [24,25,26]. Similarly, we observed apoE in the fractions with *d* = 1.063–1.021 g/mL and the fraction with *d* > 1.21 g/mL.

The existence of intact apoB in the BL medium and the comparable percentage of unesterified ^3^H-Ch between BL medium and cell-untreated lipoproteins suggest that the apoB-LPs internalized by MAECs escaped from lysosomal degradation and transcellularly resecreted. This differs from the fate of the apoB-LPs internalized by other types of cells, where internalized apoB is degraded in lysosomes [4,6]. Our data also showed that the percentage of unesterified ^3^H-Ch is significantly higher in MAECs than in cell-untreated lipoproteins. The intracellularly accumulated free ^3^H-Ch may result from an exchange of cholesterol between lipoproteins and the cell membrane.

Transport of LDLs though arterial endothelial cells is reportedly mediated by a caveolae-dependent pathway [13,16]. Our data suggest that E/B-LPs and LDLs compete with each other for transport through the MAEC monolayer, and that filipin, a caveolae inhibitor, reduced the transport of not only LDLs, but also TRLs and IDLs through MAECs. These observations suggest that apoB-LPs, whether rich or poor in apoE, share a common transport pathway, i.e., transcytosis of E/B-LPs through endothelial cells is also mediated by a caveolae-dependent pathway. Three hypotheses have been proposed to describe the caveolae-mediated transendothelial transport. One suggests that caveolae on the AP and BL membrane surface transiently fuse with each other, thereby producing channels through which the cargo passes. Another hypothesis suggests that caveolae pinch off from the plasma membrane, travel a short distance across the cell, and fuse with the opposite surface. The third hypothesis suggests that internalized caveolae, like clathrin-coated vesicles, may fuse with early endosomes. Further sorting from early endosomes may occur to recycling endosomes or lysosomes. Experimental evidence supports the possibility that all these mechanisms operate in endothelial cells [27]. The fact that apoE and apoB was distributed separately in different density fractions suggests that sorting of the constituents of E/B-LPs and LDLs may occur in MAECs, and the apoB-carrying lipid particles and HDL_E_ particles exit these cells separately. However, little is known about the itinerary of LDL- and E/B-LP-containing endocytic organelles in endothelial cells. Studies using enterocytes and Madin-Darby Canine Kidney cells suggest that early endosomes formed apically or basolaterally are directed to the same set of acidic sorting endosomes, designated as common endosomes. In these endosomes, cargos are sorted to the AP or BL recycling endosomes, followed by delivery to the corresponding plasma membranes and sequential resecretion [28]. Our data showed that radiolabeled apoE may exit the MAEC monolayer apically and basolaterally. It is likely that recycling endosomes formed in MAECs may be directed to both the AP and BL membrane. Further research is needed to explore the intracellular itinerary of LDL- and E/B-LP-containing endocytic organelles in endothelial cells.

Another important finding from this report is that the average density of the LD fractions was increased significantly in the BL media compared with the control E/B-LPs, TRLs, and IDLs. This suggests that MAECs may convert TRLs to IDLs, and IDLs to LDLs. Multiple mechanisms may be responsible for this conversion process. One of the mechanisms may be the relocation of lipids, such as cholesterol and phospholipids, from E/B-LPs to HDL_E_ particles. This lipid redistribution reduces the average size or increases the average density of E/B-LPs. In addition, hydrolysis of phospholipids and triglycerides (TGs) by endothelial lipase may also play a minor role in this conversion process. In the circulating blood, TGs in TRLs and IDLs are hydrolyzed by TG lipases, which converts TRLs to IDLs and IDLs to LDLs. The TG lipase subfamily consists of endothelial lipase, lipoprotein lipase, and hepatic lipase. Endothelial lipase is the only identified lipase that is expressed by endothelial cells. Compared with lipoprotein and hepatic lipases, endothelial lipase is relatively more active as a phospholipase than a TG lipase. Specifically, the ratio of TG lipase to phospholipase activity of endothelial lipase was 0.65, compared with ratios of 24.1 for hepatic lipase and 139.9 for lipoprotein lipase [29]. It has been demonstrated that endothelial lipase plays a role in hydrolysis of HDL phospholipids and is responsible for the reduced size of HDL particles that have passed through the endothelial cells [30]. Data are less consistent in the contribution of endothelial lipase to the metabolism of apoB-containing lipoproteins. In vitro endothelial lipase has been shown to hydrolyze phospholipids in chylomicrons, VLDLs, IDLs, and LDLs. In vivo increase in plasma endothelial lipase concentrations has been shown to be associated with a deteriorated lipoprotein-lipid profile and elevated plasma triglyceride and apoB concentrations, as well as with smaller LDL particle size [31]. Small, dense LDLs have a higher affinity for intima proteoglycans than large buoyant particles, and part of the atherogenicity of LDLs reportedly depends on their tendency to form complexes with arterial proteoglycans [32,33]. Our data showed that transendothelial transport slightly increased the average density of LDLs. This observation provided a basis for further studying the possible consequences that endothelial remodeling of apoB-LPs, especially LDL particles, could have in their interaction with the arterial intima extracellular matrix.

**Scheme 1:**
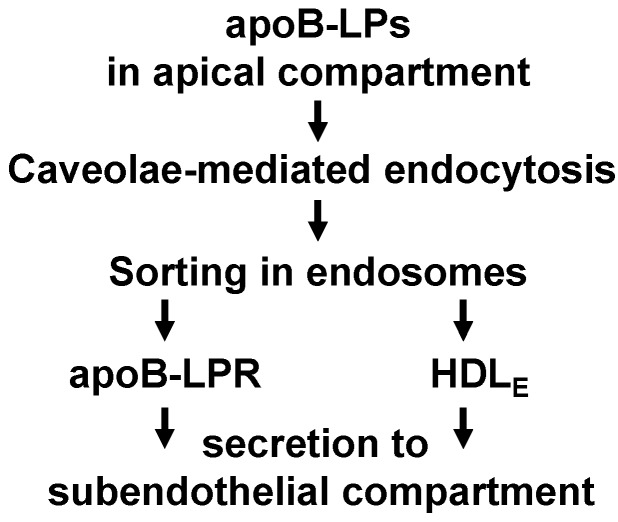
Schematic of the association of transendothelial transport of apoB-LPs with HDL_E_ formation.

In summary, data from this report suggest that transcytosis of apoB-LPs, whether rich or poor in apoE, through the MAECs generated HDL_E_ particles and converted TRLs to IDLs and LDLs. Based on the findings from the current work and the knowledge of HDL_E_ formation in other cell types [4,6], we proposed the following model to describe how transendothelial transport of apoB-LPs generates HDL_E_ formation (Scheme 1). ApoB-LPs are endocytosed from the apical membrane primarily via a caveolae-dependent pathway. The internalized apoB-LP particles disintegrate within sorting endosomes. A portion of apoE and lipids, such as cholesterol, are sorted from apoB-LPs, and forms HDL_E_. The HDL_E_ and the remodeled apoB-LP (apoB-LPR) particles are released to the subendothelial compartment through distinct trancytotic vesicles. Further studies are needed to confirm the formation and trafficking of HDL_E_ and apoB-LPR particles in endothelial cells.

## 4. Materials and Methods

### 4.1. Chemicals and Reagents

Corning™ Transwell™ Polycarbonate Membrane Inserts (07200156 and 07200170), Pierce™ Iodination Beads (PI28665), EMD Millipore™ Amicon™ Ultra-4 Centrifugal Filter Units (UFC800324), Zeba Spin 7K MWCO Desalting Columns (#89891), ECL-plus chemiluminescence reagent, and Opti-MEM™ Reduced Serum Medium were obtained from ThermoFisher Scientific (Waltham, MA, USA). [1,2-^3^H(N)]-cholesterol and ^125^-Iodine were obtained from American Radiolabeled Chemicals (St. Louis, MO, USA) and PerkinElmer (Waltham, MA, USA), respectively. Human serum and apoE3 were purchased from Biological Specialty Corporation (Colmar, PA, USA) and Leinco Technologies (St. Louis, MO, USA), respectively. Filipin (#70440) and fetal bovine serum (FBS) were purchased from Cayman Chemical (Ann Arbor, MI, USA) and Life Technologies (Carlsbad, CA, USA), respectively. Chlorpromazine (#357313) and antibodies against apoE and apoB were purchased from Santa Cruz Biotechnology, Inc. (Santa Cruz, CA, USA). Primary MAECs were purchased from Cell Biologics (Chicago, IL, USA).

### 4.2. Isolation and Radiolabeling of Lipoproteins

^3^H-Ch-labeled E/B-LPs, TRLs, IDLs, and LDLs were obtained as described by Barter et al. [34]. In brief, 300 µCi of ^3^H-Ch was incubated with 50 mL human serum containing 50 µM butylated hydroxytoluene at 37 °C for 18 h. The unassociated ^3^H-Ch was removed by incubation with an equal volume of autologous packed erythrocytes at 37 °C for 2 h. The erythrocytes were removed by centrifugation at 16,000× *g* at 4 °C for 5 min. Radiolabeled E/B-LPs (*d* ≤ 1.019), TRLs (*d* ≤ 1.006), IDLs (*d* = 1.006–1.019), and LDLs (*d* = 1.019–1.063) were isolated by potassium bromide (KBr) DG ultracentrifugation, as previously described [35]. The E/B-LPs, TRLs, IDLs, and LDLs labeled in this manner contained both esterified and free ^3^H-Ch [34]. Unlabeled E/B-LPs (uE/B-LPs), TRLs (uTRLs), IDLs (uIDLs), and LDLs (uLDLs) were isolated by KBr DG ultracentrifugation of the human serum that was not incubated with ^3^H-Ch.

For preparation of ^125^I-labeled lipoproteins, uTRLs, uIDLs, and uLDLs were incubated with ^125^I and Pierce Iodination Beads, per manufacturer’s instructions. To remove unincorporated ^125^I, the labeled lipoproteins were passed through a Zeba desalting column, and then dialyzed against 50 volumes of 0.15 M NaCl, 0.3 mM EDTA, and 10 mM Tris-HCl. For preparation of ^125^I-apoE-containing E/B-LPs and TRLs, human apoE3 was labeled with ^125^I using Pierce Iodination Beads. The ^125^I-labeled apoE was removed of free iodine by passage through a Zeba Desalting Column and then incubated with uE/B-LPs and uTRLs at a ratio of 10 μg ApoE: 1 mg lipoprotein cholesterol at 37 °C for 3 h, as described previously [36]. ^125^I-apoE E/B-LPs and ^125^I-apoE TRLs were re-isolated by KBr DG ultracentrifugation to remove free ^125^I-apoE.

### 4.3. Transendothelial Transport of apoB-LPs

MAECs were seeded in 0.4 µm transwell inserts and cultured in 6-well plates in Dulbecco’s Modified Eagle’s Medium supplemented with 10% FBS to confluence, unless otherwise stated. The AP medium was replaced with 1.5 mL of Opti-MEM™ medium supplemented with 20 µg/mL of E/B-LPs, TRLs, IDLs, or LDLs labeled with ^3^H-Ch, ^125^I, or ^125^I-apoE, while the BL medium was replaced with 2 mL of Opti-MEM^TM^ medium. In the experiments using unlabeled lipoproteins as competitors, 800 µg/mL of uE/B-LPs or uLDLs were added to the AP chamber. In the experiments using endocytosis inhibitors, the AP side of the MAEC monolayer was pretreated with 20 µg/mL chlorpromazine, 5 µg/mL filipin, or 1 µL/mL dimethyl sulfoxide (vehicle) for 1 h. After a 24-h incubation, the AP and BL media were collected. In the experiments for determination of ^125^I-apoE resecretion, the MAEC monolayer was washed thrice with 100 units/mL heparin or a low ionic strength solution, i.e., 0.2× phosphate buffer supplemented with 300 mM glycine and 5% bovine serum albumin, to remove the surface-bound materials. Fresh Opti-MEM™ medium was added into both the AP and BL chambers and incubated for another 24 h. Collected AP and BL media were centrifuged at 16,000× *g* for 10 min. The resulting supernatants and 30 µg cell-untreated radiolabeled lipoproteins were fractionated by discontinuous NaCl/KBr DG ultracentrifugation [37]. Briefly, the medium samples or radiolabeled lipoproteins were adjusted to 1.21 g/mL by addition of KBr. The discontinuous DG was constructed by sequentially layering the following solutions into a centrifugation tube: 2 mL of 1.35 g/mL KBr; 3 mL of 1.21 g/mL KBr (medium samples or radiolabeled lipoproteins); 3 mL of 1.063 g/mL KBr; 2 mL of 1.019 g/mL KBr; and 2 mL of 1.006 g/mL NaCl [37]. The constructed gradients were centrifuged at 40,000× *g* at 20 °C for 24 h using an SW-41 rotor (Beckman Coulter Life Sciences, Indianapolis, IN, USA). Twenty fractions (0.57 mL/fraction) were collected. The density of the fractions was determined by their weight, and radioactivity was determined using a PerkinElmer Tri-Carb 2300TR Liquid Scintillation counter. In the experiment where ^125^I-labeled lipoproteins and ^125^I-apoE-containing lipoproteins were used, the collected fractions were precipitated with 10% trichloroacetic acid before scintillation counting to eliminate the possible effect of free iodine.

### 4.4. Precipitation of Free Cholesterol by Digitonin

After the MAEC monolayer was incubated with ^3^H-Ch-labeled TRLs, IDLs, or LDLs as described above, the AP and BL media were collected. The MAEC monolayer was washed thrice with 100 unit/mL heparin and lysed with 0.5 M NaOH. The media and cell lysate were centrifuged at 16,000 rpm for 10 min to remove debris. The supernatants were dried using a CentriVap Concentrator (Labconco Corp., Kansas City, MO, USA). The pellet was resuspended in 500 µL ethanol containing 0.5 mg/mL unlabeled cholesterol and then mixed with 215 µL of 10 mg/mL digitonin in ethanol and 80 µL H_2_O. After an overnight incubation, the mixture was centrifuged at 16,000× *g* for 10 min. The ^3^H-Ch esters in the supernatant and free ^3^H-Ch in the pellet were determined by scintillation counting. The level of total cholesterol was calculated by the sum of ^3^H-Ch esters and free ^3^H-Ch. The percentage of free in total ^3^H-Ch (% free/total ^3^H-Ch) was calculated using the following equation: (The radioactivity in the pellet/the total radioactivity in the supernatant and in the pellet) × 100).

### 4.5. Western Blot Analysis

The MAEC monolayer was incubated with 20 µg/mL of uE/B-LPs or uLDLs in the AP side and Opti-MEM™ medium alone in the BL side for 24 h. The AP and BL media were centrifuged at 16,000× *g* for 10 min. The supernatants were fractionated by discontinuous DG ultracentrifugation as described above. Fractions 1–9 and 10–20 were pooled separately and designated as LD and HD fractions, respectively. MAECs were lysed using M-PER mammalian protein extraction reagent. Proteins in the LD and HD fractions and the cell lysate were precipitated with 75% methanol, and resolved on 4–15% SDS-PAGE gradient gels. Proteins were transferred to a PVDF membrane. After blocking with 3% fat-free milk, the membranes were incubated with antibodies against apoB and apoE. Immunoreactive bands were visualized using ECL-plus chemiluminescence reagent and analyzed with a GS-700 Imaging Densitometer (Bio-Rad Laboratories, Hercules, CA, USA) [9].

### 4.6. Statistical Analysis

Data are reported as the mean ± SEM (standard error of the mean). Differences among groups were analyzed using Student’s unpaired *t*-test (two groups) and one-way analysis of variance (more than two groups) followed by Tukey’s post-hoc test. Statistical significance was considered when *P* was less than 0.05. VassarStats software (vassarstats.net, 2015) was used for statistical analysis.
